# Human T-cell leukemia virus type I infects human lung epithelial cells and induces gene expression of cytokines, chemokines and cell adhesion molecules

**DOI:** 10.1186/1742-4690-5-86

**Published:** 2008-09-22

**Authors:** Hiromitsu Teruya, Mariko Tomita, Masachika Senba, Chie Ishikawa, Maki Tamayose, Akiko Miyazato, Satomi Yara, Yuetsu Tanaka, Yoichiro Iwakura, Jiro Fujita, Naoki Mori

**Affiliations:** 1 Division of Molecular Virology and Oncology, Graduate School of Medicine, University of the Ryukyus, 207 Uehara, Nishihara, Okinawa, Japan; 2 Division of Control and Prevention of Infectious Diseases, Graduate School of Medicine, University of the Ryukyus, 207 Uehara, Nishihara, Okinawa, Japan; 3 Department of Pathology, Institute of Tropical Medicine, Nagasaki University, 1-12-4 Sakamoto, Nagasaki, Japan; 4 Division of Child Health and Welfare, Faculty of Medicine, University of the Ryukyus, 207 Uehara, Nishihara, Okinawa, Japan; 5 The Japanese Society for the Promotion of Science (JSPS), Japan; 6 Department of Infectious Diseases and Infection Control, International Medical Center, Saitama Medical School, 1397-1 Yamane Hidaka, Saitama, Japan; 7 Division of Immunology, Faculty of Medicine, University of the Ryukyus, 207 Uehara, Nishihara, Okinawa, Japan; 8 Center for Experimental Medicine, The Institute of Medical Science, The University of Tokyo, 4-6-1 Shirokanedai, Minato-ku, Tokyo, Japan

## Abstract

**Background:**

Human T-cell leukemia virus type I (HTLV-I) is associated with pulmonary diseases, characterized by bronchoalveolar lymphocytosis, which correlates with HTLV-I proviral DNA in carriers. HTLV-I Tax seems to be involved in the development of such pulmonary diseases through the local production of inflammatory cytokines and chemokines in T cells. However, little is known about induction of these genes by HTLV-I infection in lung epithelial cells.

**Results:**

We tested infection of lung epithelial cells by HTLV-I by coculture studies in which A549 alveolar and NCI-H292 tracheal epithelial cell lines were cocultured with MT-2, an HTLV-I-infected T-cell line. Changes in the expression of several cellular genes were assessed by reverse transcription-polymerase chain reaction, enzyme-linked immunosorbent assay and flow cytometry. Coculture with MT-2 cells resulted in infection of lung epithelial cells as confirmed by detection of proviral DNA, HTLV-I Tax expression and HTLV-I p19 in the latter cells. Infection was associated with induction of mRNA expression of various cytokines, chemokines and cell adhesion molecule. NF-κB and AP-1 were also activated in HTLV-I-infected lung epithelial cells. *In vivo *studies showed Tax protein in lung epithelial cells of mice bearing Tax and patients with HTLV-I-related pulmonary diseases.

**Conclusion:**

Our results suggest that HTLV-I infects lung epithelial cells, with subsequent production of cytokines, chemokines and cell adhesion molecules through induction of NF-κB and AP-1. These changes can contribute to the clinical features of HTLV-I-related pulmonary diseases.

## Background

Human T-cell leukemia virus type I (HTLV-I) is a retrovirus responsible for adult T-cell leukemia (ATL) [1] and a chronic neurological disorder known as HTLV-I-associated myelopathy/tropical spastic paraparesis (HAM/TSP) [2,3]. HTLV-I is also implicated in several other inflammatory disorders, such as uveitis, chronic arthropathy and Sjögren's syndrome [4]. Furthermore, transgenic mice expressing Tax protein, a transactivator encoded by HTLV-I, develop proliferative synovitis [5] and exocrinopathies affecting lacrimal and salivary glands, features similar to those of Sjögren's syndrome in humans [6]. Individuals infected with HTLV-I are also known to show pulmonary involvement. For example, patients with HAM/TSP and uveitis or asymptomatic carriers frequently exhibit pulmonary complications characterized by T-lymphocyte alveolitis or lymphocytic interstitial pneumonia [7,8]. In Tax-expressing transgenic mice, inflammatory cells consisting mainly of lymphocytes accumulate in peribronchiolar and perivascular areas as well as in alveolar septa [9].

Immunological mechanisms are believed to play an important role in the pathogenesis of T-lymphocyte alveolitis in patients infected with HTLV-I, based on the cytotoxic immune response of CD8+ T cells [10], and the presence of circulating CD8+ cytotoxic T cells specific for the HTLV-I Tax in patients with HAM/TSP [11,12]. T lymphocytes, especially CD4+ T cells, are the main target of HTLV-I *in vivo *and carry the majority of the HTLV-I proviral load [13,14]. In bronchoalveolar lavage fluid of HTLV-I carriers, the copy number of HTLV-I proviral DNA correlates with the number of lymphocytes [15]. On the other hand, it has been estimated that there are 28000 type I pneumocytes, 1400 type II pneumocytes and 50 alveolar macrophages per alveolus in an average human male [16]. However, little is known about the tropism of HTLV-I for lung epithelial cells. Because HTLV-I exhibits tropism for synoviocytes, thyrocytes and retinal glial cells [17-19], we sought to determine whether lung epithelial cells can be infected with HTLV-I and whether such infection modulates the expression of cellular genes.

## Methods

### Cell culture and *in vitro *HTLV-I infection

Human A549, a type II alveolar epithelial cell line, and NCI-H292, a tracheal epithelial cell line, were maintained in RPMI 1640 containing 10% fetal bovine serum (FBS). MT-2 cells, obtained by coculture of peripheral leukemic cells from an ATL patient with normal umbilical cord leucocytes [20], were used as the HTLV-I-infected T-cell line. MT-2 cells contained proviral HTLV-I DNA and produced viral particles. CCRF-CEM cells were used as the uninfected T-cell line. These T cells were treated with 100 μg/ml of mitomycin C (MMC) for 1 h at 37°C. After washing three times with phosphate buffered saline (PBS), they were cultured with an equal number of epithelial cells in RPMI 1640 containing 10% FBS. The culture medium was changed on the third day after coculture. A549 and NCI-H292 cells were harvested at 3, 5, 8 and 14 days, followed by DNA and RNA extraction, as described below. Samples of the culture supernatant were collected at 3 and 5 days after infection and used to measure the p19 antigen of HTLV-I (ZeptoMetrix, Buffalo, NY), IL-8 (BioSource International, Camarillo, CA) and CCL20 (R&D Systems, Minneapolis, MN) by enzyme-linked immunosorbent assay (ELISA).

### RT-PCR

Total RNA was extracted with Trizol (Invitrogen, Carlsbad, CA) according to the protocol provided by the manufacturer. First-strand cDNA was synthesized from 5 μg total cellular RNA using an RNA PCR kit (Takara Bio Inc., Otsu, Japan) with random primers. Thereafter, cDNA was amplified. The sequences of the primers were described previously [18,21-30]. PCR products were fractionated on 2% agarose gels and visualized by ethidium bromide staining.

### Measurement of HTLV-I proviral load

DNA was prepared from each sample and stored at -80°C until use. The concentration of extracted DNA was adjusted to 10 ng/μl of the working solution. A quantitative real-time PCR assay was developed to measure the proviral load of HTLV-I in cells, as described previously [18].

### Immunohistochemical staining

We examined lung biopsy specimens from three patients with HTLV-I-related pulmonary diseases or normal lung biopsies, and lung biopsy specimens from transgenic mice bearing Tax or control littermate mice [9]. All subjects provided informed consent before samples were obtained. The tissue samples were subjected to immunohistochemical staining using the mouse monoclonal antibody (Ab) to Tax, Lt-4 [31]. Serial sections were deparaffinized. Antigenic sites bound by the Ab were identified by reacting the sections with a mixture of 0.05% 3,3'-diaminobenzidine tetrahydrochloride in 50 mM Tris-HCl buffer and 0.01% hydrogen peroxide. Sections were counterstained with methyl green.

### Western blot analysis

Cells were lysed in a buffer containing 62.5 mM Tris-HCl (pH 6.8), 2% sodium dodecyl sulfate, 10% glycerol, 6% 2-mercaptoethanol and 0.01% bromophenol blue. Equal amounts of protein (20 μg) were subjected to electrophoresis on sodium dodecyl sulfate-polyacrylamide gels, followed by transfer to a polyvinylidene difluoride membrane and sequential probing with the specific antibodies. The bands were visualized with an enhanced chemiluminescence kit (Amersham Biosciences, Piscataway, NJ). Mouse monoclonal Ab to actin was purchased from NeoMarkers (Fremont, CA). Mouse monoclonal Ab to Tax, Lt-4, was used.

### Flow cytometry

To measure the expression of ICAM-1 and LFA-1 on the surface of epithelial cells after HTLV-I infection, FITC-labeled mouse monoclonal Ab against ICAM-1, LFA-1 α chain or control mouse IgG1 (Coulter Immunotech Co., Marseille, France) was used. Cells were analyzed on an Epics XL flow cytometer (Beckman Coulter, Fullerton, CA) after gating on forward and side scatter to exclude debris and clumps.

### Reporter assay

A549 cells were transfected with a luciferase reporter construct for the HTLV-I long terminal repeat (LTR), and NF-κB and AP-1 reporter constructs [22,28,30] using Lipofectamine (Invitrogen). After 24 h, the transfected A549 cells were cocultured in the presence or absence of MMC-treated MT-2 or CCRF-CEM cells for 24 h before luciferase assay. Luciferase activities were measured using the dual luciferase assay system (Promega, Madison, WI) and normalized by the Renilla luciferase activity from phRL-TK.

### Electrophoretic mobility shift assay (EMSA)

EMSA was performed as described previously [22,30]. Briefly, 5 μg of nuclear extract was incubated with ^32^P-labeled probes. The DNA-protein complex was separated from the free oligonucleotides on a 4% polyacrylamide gel. For competition experiments, the cold oligonucleotide probe or competitors were used, and supershift analysis was performed using Abs against NF-κB subunits p50, p65, c-Rel, p52 and RelB, and AP-1 subunits c-Fos, FosB, Fra-1, Fra-2, c-Jun, JunB and JunD (Santa Cruz Biotechnology, Santa Cruz, CA).

## Results

### Detection of HTLV-I antigens and proviral DNA in lung epithelial cells cocultured with HTLV-I infected T cells

**Figure 1:**
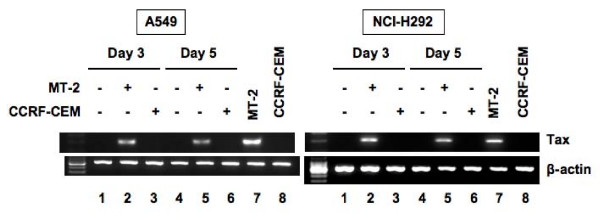
**Detection of HTLV-I Tax mRNA in A549 and NCI-H292 cells by RT-PCR**. Both cell lines were cocultured with MMC-treated MT-2 or CCRF-CEM cells. At 3 and 5 days after cocultivation, A549 and NCI-H292 cells were harvested and then Tax mRNA expression was analyzed. Human β-actin mRNA was used as a control.

A549 and NCI-H292 cells were cocultured with either MT-2 or CCRF-CEM cells. After cocultivation for 3 days, A549 and NCI-H292 cells were washed extensively and recultured in a fresh medium for another 2 days, followed by thorough washing. At 3 and 5 days post-cocultivation, A549 and NCI-H292 cells were harvested for assessment by RT-PCR for expression of HTLV-I viral antigen. Since T-cell lines were pretreated extensively with MMC, these MMC-treated T cells could not proliferate, as determined by cell proliferation assay. These specimens of A549 and NCI-H292 cells at 3 and 5 days of culture contained no viable MT-2 cells. As shown in Figure 1, A549 and NCI-H292 cells cocultured with MT-2 cells showed strong expression of Tax mRNA. In contrast, A549 and NCI-H292 cells cocultured with CCRF-CEM cells did not express Tax mRNA. Using RNA samples prepared from A549 cells cocultured with non-permissible HTLV-I-infected T cell line, TL-OmI [32], RT-PCR was carried out, but Tax mRNA was not detected (data not shown).

**Figure 2:**
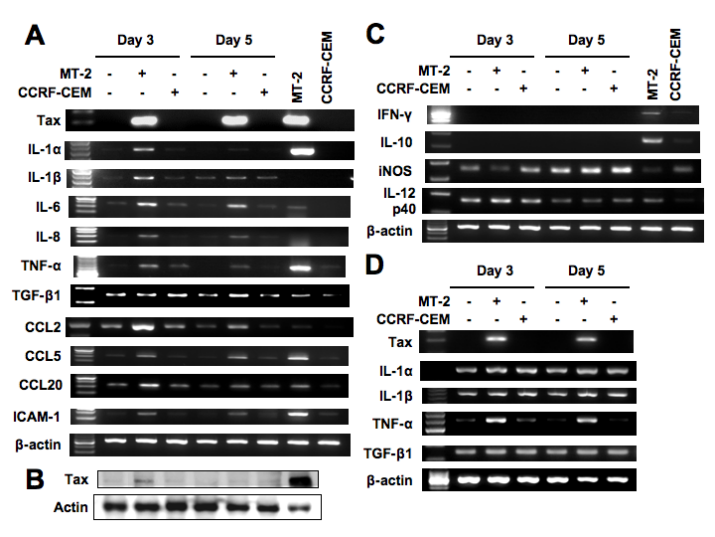
**Induction of expression of cytokines, chemokines and cell adhesion molecule in A549 cells cocultured with MT-2 cells**. A549 cells were cocultured with MMC-treated MT-2 or CCRF-CEM cells. At 3 and 5 days after cocultivation, A549 cells were harvested and then the expression of the indicated genes was analyzed by RT-PCR. (A) Genes that were upregulated by HTLV-I infection. (B) Detection of Tax protein in A549 cells cocultured with MT-2 cells. (C) Genes that were not affected by HTLV-I infection. (D) Expression of cytokine genes in NCI-H292 cells cocultured with MT-2 or CCRF-CEM cells.

We next performed Western blot analysis to assess the expression of Tax protein in A549 cells cocultured with MT-2 or CCRF-CEM cells. As shown in Figure 2B, A549 cells cocultured with MT-2 cells for 3 days expressed Tax protein. In contrast, A549 cells cocultured with CCRF-CEM cells did not express Tax protein. These results suggest that HTLV-I can be transmitted into lung epithelial cells from HTLV-I producing MT-2 cells.

To confirm the production of viral protein, A549 and NCI-H292 cells were first cocultured for 3 days either alone (control) or with MMC-treated MT-2 cells, then washed extensively and recultured in a fresh medium for 2 days. At the end of this period, the level of HTLV-I p19 core protein was measured in culture supernatants. Production of HTLV-I p19 was evident after 3 day of infection; the levels of HTLV-I p19 in the supernatants of A549 and NCI-H292 cells infected with HTLV-I were 1337 and 1023 pg/ml, respectively. The level of p19 in the supernatant of the MMC-treated MT-2 cells, the number of which corresponds to that used for coculturing, was less than 25 pg/ml. These results argue against the possibility that the p19 in the supernatant was produced by residual MT-2 cells used for infection, and support our conclusion that lung epithelial cells are infected by HTLV-I.

Using DNA samples extracted from cocultured lung epithelial cells, the pX region sequence of HTLV-I proviral DNA was amplified by real-time PCR. In A549 cells, the proviral copy numbers per 100 cells were 100, 100 and 64 at 3, 5 and 14 days, respectively. In NCI-H292 cells, the proviral copy numbers were 100, 84 and 40 at 3, 5 and 8 days, respectively. Taken together, our observations suggest that coculturing of lung epithelial cells with MT-2 resulted in infection with HTLV-I.

### Expression levels of several genes in HTLV-I-infected lung epithelial cells

Tax activates not only the transcription of the viral genome but also the expression of various cellular genes [33]. Therefore, we investigated the expression of inflammatory cytokines, chemokines and cell adhesion molecules in A549 cells cocultured with MT-2 or CCRF-CEM cells by RT-PCR. As shown in Figure 2A, the expression levels of IL-1α, IL-1β, IL-6, IL-8, TNF-α, CCL2 (MCP-1), CCL5 (RANTES), and ICAM-1 were increased in A549 cells cocultured with MT-2 cells, but not in A549 cells cocultured with CCRF-CEM cells at 3 and 5 days. The expression levels of most genes were decreased at 5 days after infection. The expression levels of TGF-β1 and CCL20 (MIP-3α) were increased in A549 cells cocultured with MT-2 cells at 5 and 3 days, respectively. Transcripts of IFN-γ and IL-10 were not detected in any of the samples. Transcripts of inducible nitric oxide synthase (iNOS) and IL-12 p40 were detected in control A549 cells, and expression levels were not different between samples (Figure 2C). Cytokine gene expression in NCI-H292 cells was also studied by RT-PCR, using cDNA samples prepared from cocultured cells. As shown in Figure 2D, high expression levels of IL-1α, IL-1β and TGF-β1 mRNA were detected in control NCI-H292 cells. However, the expression level of TNF-α was increased in NCI-H292 cells cocultured with MT-2 cells at 3 and 5 days.

The expression level of Tax mRNA was equivalent in A549 cells cocultured with MT-2 cells at 3 and 5 days, but the expression level of Tax protein was suppressed at 5 days (Figure 2B). Therefore, the expression of several cellular genes correlated with that of Tax. MT-2 cells expressed IFN-γ and IL-10 mRNA, but not IL-1β and IL-8 mRNA. However, A549 cells cocultured with MT-2 cells expressed IL-1β and IL-8 mRNA, but not IFN-γ and IL-10 mRNA, which suggests that residual MT-2 cells in these samples were not amplified.

**Figure 3:**
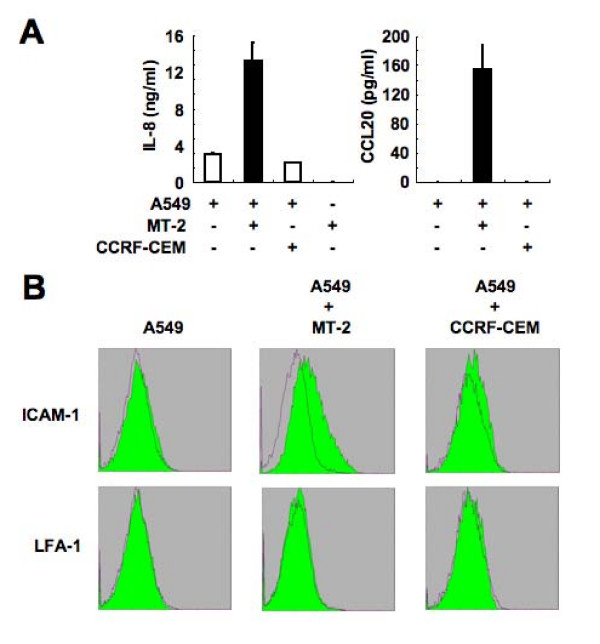
**Secretion of IL-8 and CCL20 and Induction of cell surface ICAM-1 expression in A549 cells cocultured with MT-2 cells**. (A) Secretion of IL-8 and CCL20 by A549 cells cocultured with MT-2 or CCRF-CEM cells. At 5 days after cocultivation, the levels of IL-8 and CCL20 in the supernatants were measured. Data are mean ± SD. (B) Induction of cell surface ICAM-1 expression on A549 cells cocultured with MT-2 or CCRF-CEM cells. At 5 days after cocultivation, the cell surface expression of ICAM-1 and LFA-1 was examined by flow cytometry. A549 cells were stained with FITC-labeled anti-ICAM-1, anti-LFA-1 α chain or the isotype control Ab.

We also investigated the production of IL-8 and CCL20 by A549 cells cocultured with MT-2 or CCRF-CEM cells. A549 cells cocultured with MMC-treated MT-2 cells released considerable amounts of IL-8 and CCL20 (Figure 3A). IL-8 was not detected in the media of MMC-treated MT-2 cells. We also measured the surface expression of ICAM-1 and LFA-1 on cocultured A549 cells by flow cytometry. Figure 3B shows that the fraction of cells expressing ICAM-1 but not LFA-1 was higher in A549 cells cocultured with MT-2 cells. Thus, consistent with the ability of HTLV-I to induce transcription of several cellular genes, infection of lung epithelial cells with HTLV-I increased the production of cytokines and chemokines and induced the surface expression of cell adhesion molecule.

### Activation of NF-κB and AP-1, and viral promoter LTR in HTLV-I-infected lung epithelial cells

**Figure 4:**
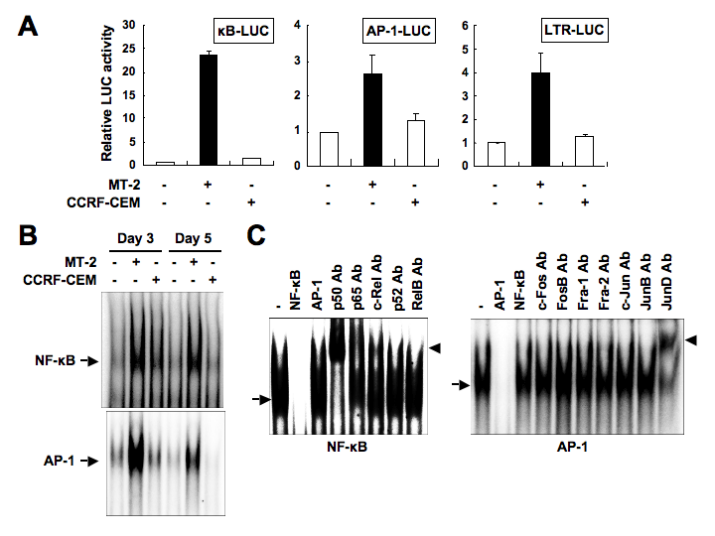
**Activation of transcription factors NF-κB and AP-1, and viral promoter LTR in A549 cells cocultured with MT-2 cells**. (A) A549 cells were transfected with κB-LUC, AP-1-LUC or LTR-LUC. After 24 h, transfected A549 cells were cocultured with MMC-treated MT-2 or CCRF-CEM cells for 24 h before luciferase assay. Luciferase activity is presented as a fold induction relative to the basal level measured in A549 cells that were not cocultured with MT-2 or CCRF-CEM cells. Relative luciferase amounts were normalized to equivalent Renilla expression to control for transfection efficiency. Data are mean ± SD values of three independent experiments. (B) DNA-binding activities of NF-κB and AP-1 in A549 cells. A549 cells were cocultured with MMC-treated MT-2 or CCRF-CEM cells. At 3 and 5 days after cocultivation, A549 cells were harvested and then NF-κB and AP-1 DNA-binding activities were analyzed by EMSA. Specific bands are indicated by arrows. (C) Characterization of DNA-protein complexes present in nuclei of A549 cells cocultured with MT-2 cells. At 5 days after cocultivation, nuclear extracts were prepared from A549 cells. The competitors used were the NF-κB site of the IL-2 receptor α chain gene and the AP-1 site of the IL-8 gene. Supershift assay in the same nuclear extracts was also performed. Supershifted bands with Abs are indicated by arrowheads.

Tax activates the expression of various cellular genes through the NF-κB and AP-1 pathways [33]. Therefore, we investigated the transcriptional activity of NF-κB and AP-1. A549 cells cocultured with MT-2 cells exhibited high transcriptional activity of NF-κB and AP-1, compared with control A549 cells and A549 cells cocultured with CCRF-CEM cells (Figure 4A). Furthermore, viral promoter LTR was activated in A549 cells cocultured with MT-2 cells, suggesting that A549 cells were infected with HTLV-I. Next, we examined DNA-binding of NF-κB and AP-1. A549 cells were cocultured with MMC-treated MT-2 or CCRF-CEM cells, and the DNA-binding activity of NF-κB and AP-1 was assessed by EMSA. As evidenced from Figure 4B, coculture of A549 cells with MT-2 induced the DNA-binding of NF-κB and AP-1. These complexes were due to specific binding of nuclear proteins to each sequence because the binding activities were reduced by the addition of cold probe but not by an irrespective sequence. This gel shift assay detected an NF-κB complex that was supershifted by anti-p50, anti-p65 and anti-c-Rel Abs, and an AP-1 complex that was supershifted by anti-JunD Ab as was noted in HTLV-I-infected T-cell lines [34,35] (Figure 4C).

### Detection of Tax protein in the lungs of Tax transgenic mice and patients with HTLV-I-related pulmonary diseases

**Figure 5:**
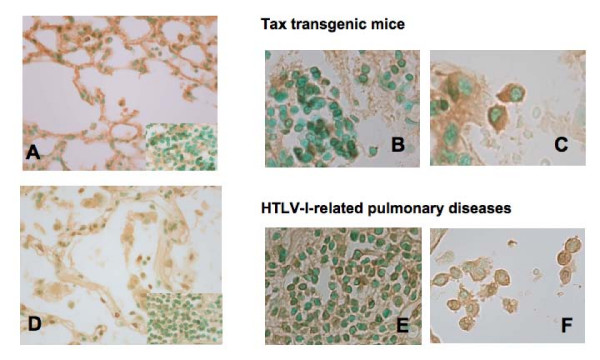
**Detection of Tax protein by immunohistochemistry**. In lung tissues of Tax transgenic mice (A-C) and patients with HTLV-I-related pulmonary diseases (D-F), immunohistochemical staining showed definite brownish staining for Tax protein in the cytoplasm of epithelial cells (A and D), and infiltrated lymphocytes (B and E) and macrophages (C and F).

Finally, we immunostained lung tissues of transgenic mice to assess the expression of viral antigen Tax. Lymphocytes accumulated in alveolar septa of the lungs of transgenic mice (Figure 5A; right lower panel), but not in littermate mice (data not shown). We examined the distribution of Tax protein in the lungs of transgenic mice. Marked expression of Tax was observed in epithelial cells (Figure 5A) as well as lymphocytes (Figure 5B) and macrophages (Figure 5C) in the lungs of transgenic mice. We next immunostained lung tissues obtained from patients with HTLV-I-related pulmonary diseases. Compatible with the lungs of HTLV-I-related pulmonary diseases, accumulation of lymphocytes was noted in alveolar septa (Figure 5D; right lower panel). Tax expression was noted in epithelial cells (Figure 5D), lymphocytes (Figure 5E) and macrophages (Figure 5F) of patients with HTLV-I-related pulmonary diseases, but not those of normal lungs (data not shown).

## Discussion

We obtained evidence that lung epithelial cells can be infected by HTLV-I and that this infection induced several genes expression. By coculturing A549 and NCI-H292 cells with the MT-2 cell line, HTLV-I proviral DNA was detected from 3 days to 2 weeks. Demonstration of expression of viral Tax at both mRNA and protein levels, and production of a viral antigen p19 in the supernatant also provided supportive evidence that HTLV-I infection and viral gene expression had taken place in lung epithelial cells. The proviral copy numbers showed a gradual decrease after infection. This transient infection was noted in retinal glial cells [19]. The possibility that HTLV-I-infected lung epithelial cells do not produce a large amount of virus and show a slower growth rate was raised by a report of Sato et al [19]. Recent data have indicated that HTLV-I infection leads to arrest in G_1 _phase of the cell cycle and senescence [36,37]. Another possibility is that HTLV-I infection might have induced apoptosis of infected cells, hence, elimination of the infected cells [19].

In this study, HTLV-I Tax was detected in lung epithelial cells from patients with HTLV-I-related pulmonary diseases and Tax transgenic mice. This finding indicates that HTLV-I can be transmitted into lung epithelial cells from infected T cells and the integrated HTLV-I genes can be transcribed and expressed.

Lung epithelial cells produce a variety of cytokines and chemokines that regulate the immune system. They also function as an important sentinel system against pathogens. The pathogenic significance of aberrant production of inflammatory cytokines and chemokines in the lung has been given increasing attention and a variety of cytokines and chemokines are considered to play important roles in the pathogenesis of lung inflammatory diseases. Lung epithelial cells cocultured with MT-2 cells expressed the mRNAs of IL-1α, IL-1β, IL-6, IL-8, TNF-α, TGF-β1, CCL2, CCL5, CCL20 and ICAM-1, but not IFN-γ, IL-10, iNOS and IL-12 p40. The expression of IL-1α, IL-1β, IL-6, IL-8, TNF-α, CCL2, CCL5 and CCL20 is regulated by NF-κB [22,24,30,38]. Furthermore, IL-8 and TGF-β1 are AP-1 targets [30,39]. In contrast, the expression of IL-10 is mediated by STAT [40]. There are many putative transcription factor-binding sites such as AP-1, GATA, NF-AT and ATF in the promoter of IFN-γ gene and they play a key role in the transcription of IFN-γ gene [41]. NF-κB, STAT, AP-1, C/EBPβ and β-catenin/TCF4 are important transcription factors in regulation of iNOS expression [42]. Furthermore, NF-κB, Ets, C/EBPβ, Sp1 and AP-1 contribute to the regulation of IL-12 p40 expression [43]. Because the expression of genes that were induced in A549 cells cocultured with MT-2 cells was regulated by only NF-κB and AP-1, and Tax protein increases transcription of cellular genes through NF-κB and AP-1, [33] we examined the activities of NF-κB and AP-1 in these cells. As expected, these cells exhibited aberrant activation of NF-κB and AP-1. These findings suggest that Tax protein could induce the transcription of cytokines, chemokines and cell adhesion molecules in lung epithelial cells in a manner similar to that in HTLV-I-infected T cells. The results of the present study suggest that lung production of inflammatory cytokines and chemokines by HTLV-I-infected epithelial cells, in addition to that by infiltrating lymphocytes, may also play a role in the pathogenesis of HTLV-I-related pulmonary diseases. To confirm this hypothesis, further studies are necessary to carry out a histopathological and molecular analysis study using lung specimens of Tax transgenic mice and patients with HTLV-I-related pulmonary diseases.

## Conclusion

It has been reported that immunoregulatory disturbances caused by HTLV-I infection can cause inflammatory multisystem diseases, including uveitis, chronic arthropathy and Sjögren's syndrome [4], in addition to the HAM/TSP [2,3] and T-lymphocyte alveolitis or lymphocytic interstitial pneumonia [7,8]. The pathological association of HTLV-I with inflammatory multiorgan diseases in HTLV-I carriers still remains to be clarified. Our study, however, suggests that the variety of clinical syndromes may be attributed to the cell tropism of HTLV-I and distribution of HTLV-I-affected cells in various organs. In summary, lung epitheial cells may be infected by HTLV-I. This process promotes the production of inflammatory cytokines and chemokines, and the expression of cell adhesion molecules by the infected cells. Such process may be involved in the pathogenesis of HTLV-I-related pulmonary diseases.

## Competing interests

The authors declare that they have no competing interests.

## Authors' contributions

HT designed the study, and performed the analysis. MS performed immunohistochemical staining. MTo and CI collected and assembled the data. MTa, SY, AM and JF provided lung biopsy specimens. YT and YI provided the antibody and the Tax-expressing transgenic mice, respectively. NM made substantial contributions to the conception and design of the study, wrote and drafted the manuscript, and contributed to data interpretation. All authors read and approved the final manuscript.
